# An Optimization Study of Estimating Blood Pressure Models Based on Pulse Arrival Time for Continuous Monitoring

**DOI:** 10.1155/2020/1078251

**Published:** 2020-02-10

**Authors:** Jiang Shao, Ping Shi, Sijung Hu, Yang Liu, Hongliu Yu

**Affiliations:** ^1^Institute of Rehabilitation Engineering and Technology, University of Shanghai for Science and Technology, Shanghai 200093, China; ^2^Wolfson School of Mechanical, Electrical and Manufacturing Engineering, Loughborough University, Ashby Road, Loughborough, Leicestershire LE11 3TU, UK; ^3^Department of Cardiovascular Surgery, Changhai Hospital, Second Military Medical University, Shanghai 200433, China

## Abstract

Continuous blood pressure (BP) monitoring has a significant meaning for the prevention and early diagnosis of cardiovascular disease. However, under different calibration methods, it is difficult to determine which model is better for estimating BP. This study was firstly designed to reveal a better BP estimation model by evaluating and optimizing different BP models under a justified and uniform criterion, i.e., the advanced point-to-point pairing method (PTP). Here, the physical trial in this study caused the BP increase largely. In addition, the PPG and ECG signals were collected while the cuff bps were measured for each subject. The validation was conducted on four popular vascular elasticity (VE) models (MK-EE, L-MK, MK-BH, and dMK-BH) and one representative elastic tube (ET) model, i.e., M-M. The results revealed that the VE models except for L-MK outperformed the ET model. The linear L-MK as a VE model had the largest estimated error, and the nonlinear M-M model had a weaker correlation between the estimated BP and the cuff BP than MK-EE, MK-BH, and dMK-BH models. Further, in contrast to L-MK, the dMK-BH model had the strongest correlation and the smallest difference between the estimated BP and the cuff BP including systolic blood pressure (SBP) and diastolic blood pressure (DBP) than others. In this study, the simple MK-EE model showed the best similarity to the dMK-BH model. There were no significant changes between MK-EE and dMK-BH models. These findings indicated that the nonlinear MK-EE model with low estimated error and simple mathematical expression was a good choice for application in wearable sensor devices for cuff-less BP monitoring compared to others.

## 1. Introduction

Increased aortic stiffness in hypertensive individuals is a fundamental manifestation of longstanding hypertension-related damage that stiffens the large arteries [1]. Uncontrolled hypertension or high blood pressure (BP) is a major risk factor that links to the potential development of serious diseases such as stroke, hypertensive heart disease, and coronary artery disease [2]. BP is influenced by many factors such as food, exercise, mental situations, and stress, among others; thus, it varies considerably from time to time [3]. Instantaneous information about BP status can be obtained from conventional standard cuff-based BP measurements, such as oscillometry [4] and auscultation. However, the above methods are not applicable to ambulatory BP monitoring (ABPM) or home BP monitoring (HBPM) due to the population-averaged nature of the BP estimation algorithm [5] and the limited frequency of measurement [6]. For cuff-based approaches, recurrent inflating and deflating of the cuff stress the patient, which causes periodic interruptions to blood flow, affecting the physiological state of the patient and disturbing the quality of sleep [7]. Moreover, cuff measurements are occlusive, cumbersome, provide only intermittent BP readings, and do not readily extend to low-resource settings [1, 3]. Hence, cuff-less continuous BP monitoring has received much attention due to its comfort and convenience compared to cuff-based approaches. Moreover, the cuff-less solution has a promising application prospect for continuous noninvasive BP monitoring by virtue of overcoming disturbance issues existing in the traditional cuff-based method [8, 9]. Photoplethysmography (PPG), a noninvasive optical measurement technique by means of photoelectric measurement, obtains physiological signals and characteristics of the human body by detecting changes in blood volume in microvessels. PPG is also a feasible technology for cuff-less continuous BP monitoring, especially in surgery, and can provide valuable information on physiological heart monitoring and cardiovascular system assessment of vascular parameters [10, 11].

Pulse arrival time (PAT) is defined as a time interval between *R*-peak and the point with maximum gradient on the rising edge of the PPG [8] which is a noninvasive optic-electrical signal. The PAT in PAT-based BP measurement can be simply measured from electrocardiography (ECG) and from PPG by wearable devices [12, 13] or a contactless video camera [14]. More importantly, PAT is dependent on both ventricular contraction and vascular function [15]. Thus, it has been commonly used as an indicator to cufflessly and continuously estimate BP under various BP changing conditions.

In the past few years, several studies reported that PAT has shown a high correlation with BP, especially systolic blood pressure (SBP) [8, 11, 16]. Some studies also investigated the potential of PAT-based measurement as a surrogate for cuff BP under different protocols [17, 18]. Advances in dynamic monitoring technologies have reinforced these impressions, especially for wearable technologies [4, 9, 19, 20]. For example, Bilo et al. designed a wearable device (Somnotouch NIBP) to evaluate its accuracy for noninvasive continuous BP monitoring using PAT according to the European Society of Hypertension International Protocol [19]. Pandian et al. developed a smart vest, which used an array of sensors connected to a central processing unit with firmware for continuously monitoring physiological signals including ECG, PPG, and BP [20]. Similarly, Zheng et al. proposed an armband wearable system, which was evaluated against a standard cuff-based device on both healthy and hypertensive subjects over a 24 h period for potential use in hypertensive management [9]. Tang et al. also developed a chair-based unobtrusive monitoring system that estimates BP using PAT calculated from ECG and PPG signals for facilitating long-term HBPM [4]. Furthermore, the methods of neural network or machine learning were paid more attention during investigating these PAT-based approaches for BP modeling [20]. Although the BP monitoring solutions described above were helpful, their accuracy of estimated BP methods, such as linear estimation [21], nonlinear estimation [4, 9, 22, 23], and regression approaches [24, 25], is still needed to improve to meet the association for the Advancement of Medical Instrumentation (AAMI) standard [26].

Most importantly, the PAT-based BP estimation method needed an individualized calibration procedure to obtain unknown coefficients or parameters in the BP estimation model for each subject before BP monitoring. The model's parameters determined after initial calibration will not change in the process of BP estimation. It was well known that different calibration methods made the model showing different performance [27]. In order to show which model had better performance, it was necessary to employ a comparison study under the same calibration mode. The least-square method (L-S) and point-to-point pairing method (PTP) were usually employed to determine the calibration parameters for BP estimation. For the L-S method, sample numbers were directly related to the accuracy of the BP estimation model. It was difficult to explain how large sample numbers were needed to meet long-term ABPM and HBPM [28]. For example, Nabeel et al. recruited 32 subjects for the calibration of the BP estimation model [22] and Esmaili et al. collected 35 subjects for the calibration of their model [29]. They largely limited BP estimation's practical application. Comparing with the L-S method, PTP required only a small initial sample number for the calibration of the estimation model. At present, there were many reports that only one sample (point) was required to calibrate the specific model [4, 9], and the model's parameters determined after initial calibration will not change in the process of BP estimation. Therefore, this paper used PTP as the BP estimation model's calibration method and further optimized this method.

There was no doubt that it was essential to study and improve the accuracy of cuff-less BP estimation models and the simplicity of calibration methods for providing a more practical solution to achieve long-term ABPM and HBPM. To date, under a justified criterion, no investigations have conducted comparative and optimal studies on the PAT-based BP estimation to reveal a better BP estimation method with both simplicity and accuracy. In the present study, five representative BP-PAT models, under a uniform criterion, were analyzed and optimized to work out which model was accurate and fitted well in continuous cuff-less BP monitoring based on a cardiovascular mechanism. This study also offered insights into future research in ambulatory cuff-less BP estimation.

## 2. Mathematical Models

Electromechanical coupling in the heart causes blood to eject into the whole arterial tree. This physiological process affects the velocity of blood flow and generates systemic pressure waves from the central to the peripheral artery. The velocity of this pressure pulse is determined by the elastic and geometric properties of the arterial wall and the blood density. The central arteries push blood to narrow distal arteries under the pressure of circulating blood on the walls of blood vessels, causing the phenomenon that the heart expands during systole and contracts during diastole. Here, the circulatory pressure is BP. Arterial BP, as a hemodynamic parameter, fluctuates on a beat-to-beat basis due to the dynamic interplay from vasomotion, neural regulation, and arterial mechanisms [30]. Physiologically, it is affected by four factors: arterial compliance, cardiac output, peripheral resistance, and blood volume [31].

Given the fluid is contained in an elastic conduits system, energy is transmitted predominantly in the arterial wall rather than through the in-compressible blood. The material characteristics, thickness, and lumen diameter of the arterial wall thus become the major determinants of the pressure wave velocity (PWV). Considering that the Moens–Korteweg (M-K) equation [8] models a relationship between the wave speed or pulse wave velocity (PWV) and the incremental elastic modulus (a coefficient of elasticity) of the arterial wall or its distensibility [32], VE models are built on this basis. Combining it with an exponential arterial elasticity model [21, 33], a new BP-PAT model, called the MK-EE model, will be obtained. It gives a logarithmic relationship between BP and the PAT. For the MK-EE model, assuming there is a negligible change in the arterial thickness and diameter with pressure variations, BP and the PAT can be linearly related by differentiating the M-K equation, called the L-MK model [21]. To overcome the bad linear correlation of DBP in the L-MK model, the Bramwell–Hill (B-H) equation [9] is introduced in estimating BP to make it have a high correlation, which is called the MK-BH model [4]. Recently, Poon et al. established a mathematical relationship between MBP and a factor that the change in elasticity is caused by pressure wave variations. It could be regarded as the development model of MK-BH, called the dMK-BH model [9].

**Figure 1:**
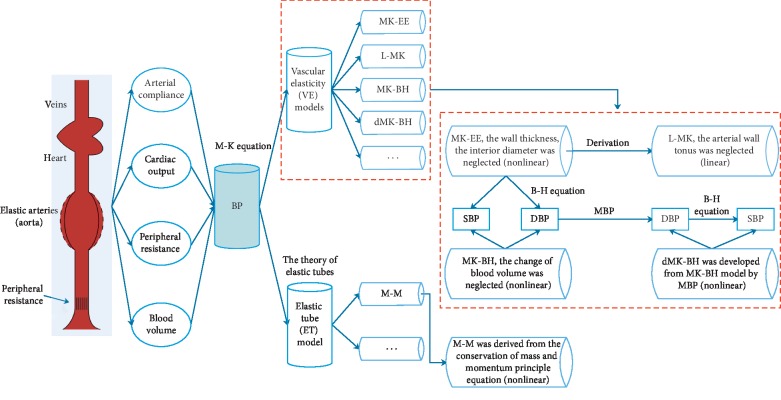
The relationships between the BP estimation models.

In recent years, models used for estimating BP based on PAT and capturing BP variations indirectly mainly fell into two categories: vascular elasticity (VE) models and elastic tube (ET) model. ET models were built on the theory of elastic tubes. It was noted that the blood flow in the arteries could be modeled as the propagation of pressure waves inside elastic tubes. A novel BP estimation nonlinear model was derived from the conservation of mass and momentum principle equation, called the M-M model [29]. Understanding the internal relationship between VE models and ET models was necessary. The principles of their modeling were shown in Figure 1.

The mathematical relationships between BP and the PAT reported in the literature were summarized in Table 1.

## 3. Methods

### 3.1. Hardware and Parameter Identification

**Figure 2:**
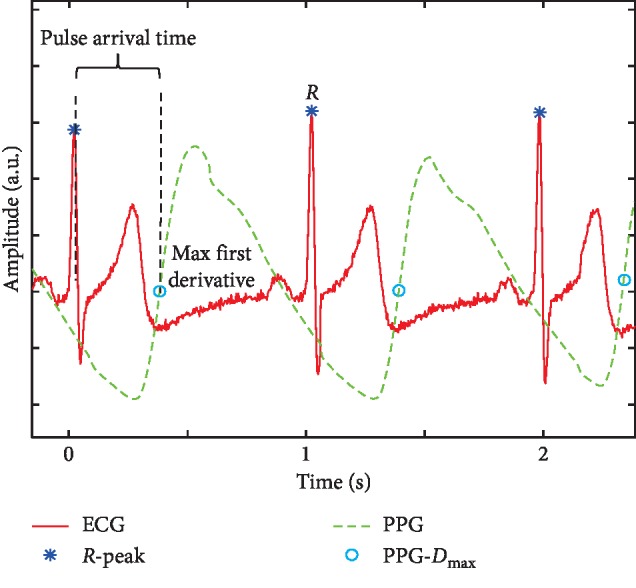
Example of PAT delineation.

In the experiments, the PowerLab/16sp system (Castle Hill, AD Instruments, Australia, 2002) was used to synchronously record and amplify the ECG and PPG signals. The ECG signal was filtered by a 1 Hz high-pass filter and a 40 Hz low-pass filter. Meanwhile, the PPG signal was filtered by a 0.5 Hz high-pass filter and a 20 Hz low-pass filter, and the sampling frequency was 1 kHz [24]. To obtain the PAT parameter, the ECG signal was employed as the proximal timing reference, and the PPG signal was used as the distal timing reference. The PAT was calculated as the time elapsed from the *R*-peak of the ECG signal to the maximum of the first derivative of the PPG wave within the same cardiac cycle in Figure 2.

### 3.2. Data Acquisition Procedure

Twelve subjects without a history of cardiovascular or neurological disorders participated in this study (see Table 2). All participants gave written informed consent. The study was approved by the health center authorities at the University of Shanghai for Science and Technology.

**Figure 3:**
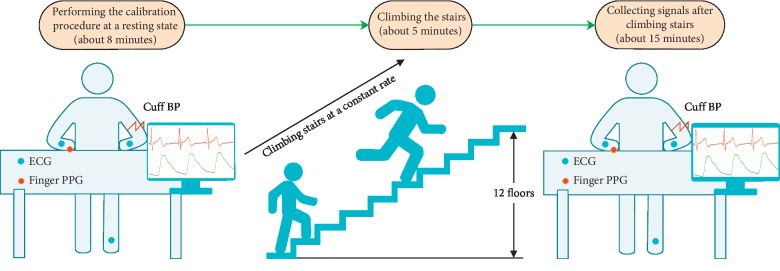
Illustration of the experimental design and the data collection procedure.

Each subject was required to climb 12 floors at a constant rate lasting for five minutes, which guaranteed a greater change in BP to obtain more accurate model estimation [15]. When the physical exercise was finished, each subject was asked to sit upright and measure the cuff BP, the ECG signal, and the PPG signal. Each subject was asked to sit on a chair 25 cm away from the table, with the cuff wrapped around his/her right arm at the same level between its center and the subject's heart. The PPG sensor was placed on their left hand to avoid the effect of cuff inflation on the PPG signals [4] (see Figure 3).

During the data collection, carrying mobile phones and wearing devices were not allowed. Moreover, subjects were also required to remain stable and breathe naturally to avoid motion causing interference from the collected signals. The data collection took approximately 15 minutes for each subject. The measured BPs generally decreased. Hence, the continuous ECG and PPG signals were collected to calculate a series of continuous PATs ($\bar{\text{PATs}}$).

### 3.3. Data Analysis

#### 3.3.1. The Advanced PTP Method: A Justified and Uniform Criterion

An automatic digital BP monitor device (MB-300C, Jasun, China) was used to measure cuff BP values. Based on its manufacturer's introduction manual, the accuracy was approximately 3 mm·Hg, which was in compliance with the conventional health standards [26]. For instance, the mean absolute error (MAE) of less than 5 mm·Hg was considered to be the acceptable maximum error according to the AAMI guidelines [26]. In addition, prior to the data collection process, we randomly measured the BP for six subjects using both an MB-300C Jasun device and a conventional mercury sphygmomanometer operated by a professional nurse with the rigorous experimental process to carry out contrast verification. During BP measurement using both monitors, the measured BPs for each subject at the rest condition were approximately the same as expected. Specifically, the mean absolute errors (MAEs) of SBP and DBP measurements using these two devices were 2.7 and 3.2 mm·Hg for six subjects, respectively.

**Figure 4:**
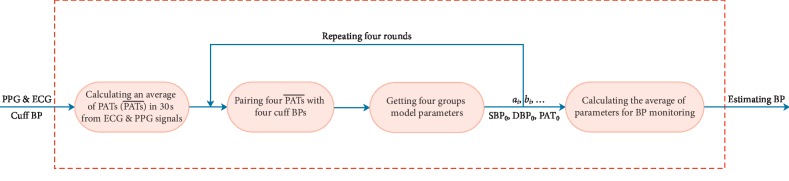
The advanced PTP method for the BP monitoring system.

Considering that it took 30 seconds to measure one cuff BP with MB-300C, an average value of PATs in these 30 seconds [22] from corresponding PPG and ECG signals was calculated to estimate BP based on its estimation model. The mapping relationship between the dependent variable and the independent variable was established through the available initial values. This technique was called the PTP method, i.e., cuff BP vs. $\bar{\text{PATs}}$. Recently, the PTP method [4, 9, 30] only used one point (sample) to calibrate the parameters of the corresponding BP estimation model. In fact, such a single point (sample) played a vital role in the BP estimation models. Consider the truth that the subject's BP was not a constant even in a quiet or peace state; therefore, it was necessary to average and balance this quiet and peace process. In this study, a new calibration method was proposed, which was shown in Figure 4.

Once the parameters of the BP estimation model were determined, they would not be changed in the estimating BP process. In the advanced PTP method, four pairs of cuff BP vs. $\bar{\text{PATs}}$ were collected to obtain four sets of parameters of BP estimation models. For example, the M-M model only needed to select three in the four pairs of cuff BP vs. $\bar{\text{PATs}}$ to complete one round calibration and got four (*C*_4_^3^) group model parameters. Given the possibility that cuff BP values of subjects in quiet state were the same, four rounds of calibration procedure were repeated to guarantee the validity of calibration in the present study. Finally, the average values of these parameters were taken as the final BP monitoring parameters, i.e., SBP_0_,  DBP_0_,  PAT_0_, *a*_*i*_,  *b*_*i*_ . The whole calibration process took about eight minutes, and the subjects were required peace and quiet state.

For the ET model, three cuff BPs were used with one round calibration. For the VE models, the MK-BH and dMK-BH required one cuff BP, and the L-MK and MK-EE needed two cuff BPs with one round calibration. The calibration was done only one time for each subject, and after deriving parameters in the BP estimator model, the BP could be estimated continuously.

#### 3.3.2. Data Test

In the present experiment, over 30,000 heartbeats were analyzed. Moreover, about 3,000 heartbeats were studied for each subject. The estimated BP from a 30 s period of ECG and PPG signals was calculated. To avoid the effect of breathing, at least eight cardiac cycles [34] were used for calculating the average value of $\bar{\text{PATs}}$. A total of 365 pairs of valid $\bar{\text{PATs}}$ vs. BPs were found, and 30 $\bar{\text{PATs}}$ during the 15-minute experiment were applied in the BP estimation for each subject.

The estimated errors between the cuff BP and the estimated BP were evaluated as the mean error (ME) ± standard deviations (SD) as well as the mean absolute difference (MAD), which were defined below:

(1) $$\begin{array}{c} \text{ME}=\frac{1}{n}\overset{n}{\underset{i=1}{\sum }}\left({\text{BP}}_{{\text{est}}_{i}}-{\text{BP}}_{{\text{cuf}}_{i}}\right),\\ \text{MAD}=\frac{1}{n}\overset{n}{\underset{i=1}{\sum }}\left|\left({\text{BP}}_{{\text{est}}_{i}}-{\text{BP}}_{{\text{cuf}}_{i}}\right)\right|,\\ \text{SSE}=\overset{n}{\underset{i=1}{\sum }}{\left({\text{BP}}_{{\text{est}}_{i}}-{\text{BP}}_{{\text{cuf}}_{i}}\right)}^{2},\\ \sigma =\sqrt{\frac{\text{SSE}}{n-1}},\\ \text{c.v.}=\frac{\left|\sigma \right|}{\text{ME}},\\ \text{SD}=\sqrt{\frac{1}{n}\overset{n}{\underset{i=1}{\sum }}{\left(x-\bar{x}\right)}^{2}}, \end{array}$$

where BP_est_*i*__ and BP_cuf_*i*__ denoted the  *i*th BP measured through BP estimation models and by the reference cuff method, respectively, and *n* was the number of measured BP used for evaluation. Further, the *x*_*i*_ denoted the *i*th error sample.

## 4. Results

The merits of the BP model based on PAT were evaluated from three different analytical methods, i.e., correlation analysis, performance analysis, and statistical analysis.

### 4.1. Correlation Analysis

The correlations between estimated BPs (BP_est_) and cuff BPs (BP_cuf_) for five models were shown in Table 3.

As shown in Table 3, the correlation between estimated SBP and cuff SBP was stronger than that between estimated DBP and cuff DBP during each nonlinear PAT-based BP monitoring model. The L-Mk model, as a linear VE model, had the weakest correlation between both SBP (*R* = 0.5537) and DBP (*R* = 0.6837) than other nonlinear BP estimation models. For MK-BH and M-M, the correlation between the cuff BP and the estimated BP was weak with a correlation coefficient of *R*_#3_ = 0.8131 for SBP and *R*_#5_ = 0.7651 for DBP. In our experiments, MK-EE and dMK-BH had a higher correlation between both SBP and DBP than other models. dMK-BH had the highest correlation between both SBP (*R* = 0.8873) and DBP (*R* = 0.8611) among all subjects.

### 4.2. Performance Analysis

To optimize and analyze the performance of the five popular models, the dispersion degree, overall comparison, and sensitivity analysis were analyzed in this study.

#### 4.2.1. Dispersion Degree

**Figure 5:**
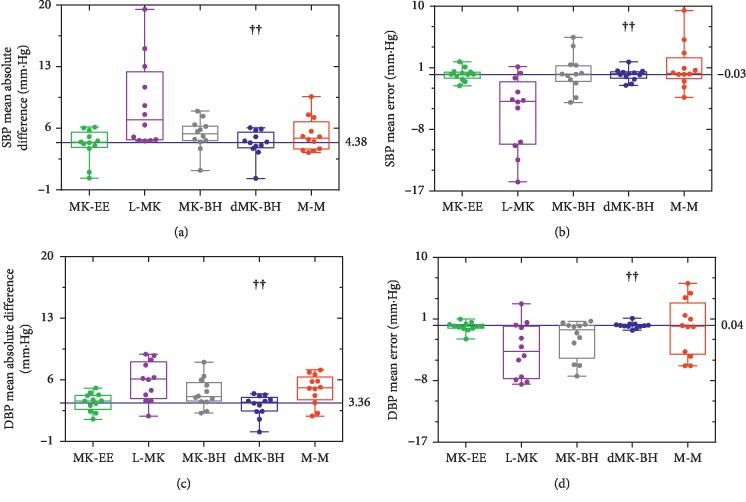
Box plots of dispersion degree comparison during different estimation methods in (a), (b) and (c) (d). Box and whisker plots: box, first and third quartiles; horizontal line, median; whiskers, the furthest point that lies no more than 1.5 times the interquartile range from the median. Note: each point represented an independent subject (estimated error). The dotted blue line represented the median value for dMK-BH (the strongest correlation, see Table 3). “**††**” indicated the BP estimation model with the strongest correlation.

A good BP estimation model should be widely applicable, not only for a few individuals to show better performance. Based on this, the estimation errors of all subjects were analyzed and compared under the same BP-PAT model. The box plot of the dispersion degree comparison was shown in Figure 5 to indicate different levels of estimated BP quality for five popular BP-PAT models.

In Figure 5, the L-MK model showed the largest MAD and ME for BP and SBP, respectively. The dMK-BH model showed the minimum median MAD of SBP with 4.38 mm·Hg and a MAD of DBP with 3.36 mm·Hg, while the ME of BP was more scattered than others in the M-M model.

As mentioned above, the great changing range of BP benefited the BP estimation for each subject. Therefore, the subject with the largest range for cuff BP (SBP: 137.19 ± 12.02 mm·Hg and DBP: 84.14 ± 5.56 mm·Hg) was further investigated to estimated BP on five models quantitatively during this research. The summed square of residuals (SSE) and the root mean square error (RMSE) were computed to report how much difference between estimated BPs and cuff BPs in this subject. The results were reported in Table 4.

**Figure 6:**
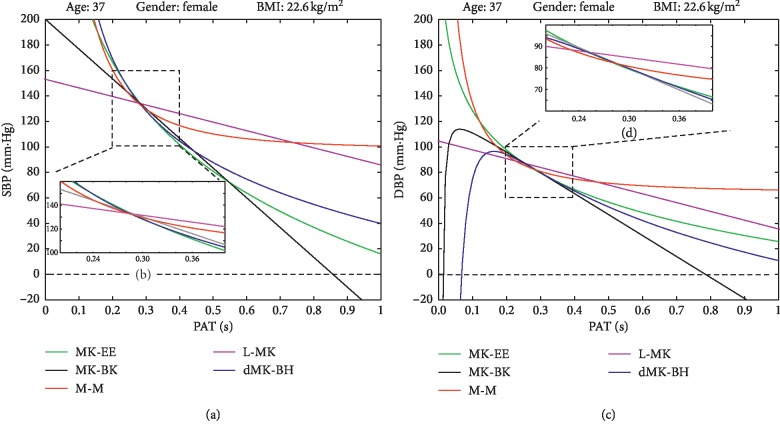
The five BP-PAT function curves of the subject with the largest BP range: (a) SBP trend in general. (b) SBP trend in the experiment. (c) DBP trend in general. (d) DBP trend in experiment.

In Table 4, it could be observed that SSE and RMSE were largest in L-MK. Moreover, this model had the smallest CV for BP estimation, while the dMK-BH model had the opposite performance as the L-MK. The M-M model, as a ET model, had larger SSE and RMSE and smaller CV than others for SBP and DBP estimation. It also could be found that the CV values were immensely different on SBP and DBP estimation for five models in this subject. Further, their function curves were shown in Figure 6.

As shown in Figure 6, the five models showed to be quite different. The MK-BH was not a bound function and not always maintains positive, which made no sense that BP varies in a negative and infinite range in Figure 6(a). Additionally, the L-MK was inconsistent with the downward trend of others in SBP and DBP estimation based on the experiment data. The MK-EE model and dMK-BH model showed the closest estimated BP performance between SBP and DBP estimation. It was worthy of note that the M-M model converged prematurely although it was a bound function. In other words, it was not insistent with the real condition due to little changed BP estimation with the increase of PAT.

#### 4.2.2. Overall Comparison

Another criterion for performance evaluation included ME, MAD of estimation, and SD of estimation during five BP estimation models was shown in Table 5.

It could be found that the L-MK model had a mean ± SD (MAD) of −5.89 ± 12.74 (9.34) mm·Hg for SBP and −3.72 ± 6.79 (5.91) mm·Hg for DBP, respectively. The dMK-BH model had a mean ± SD (MAD) of −0.01 ± 5.90 (4.55) mm·Hg for SBP and 0.04 ± 4.40 (3.38) mm·Hg for DBP, respectively. The M-M model had a mean ± SD (MAD) of 1.11 ± 7.51 (5.57) mm·Hg for SBP and −0.23 ± 6.47 (5.13) mm·Hg for DBP estimated error, respectively. For the dMK-BH model, the precision of estimation was approximately 0.06 mm·Hg higher than that of MK-EE and approximately 0.1–6 mm·Hg higher than all the other comparison methods. Among the optimized methods, both MK-EE and dMK-BH worked best for the estimated BP_cuf_ value due to the SD of the error bias being less than 5.90 mm·Hg and MAD being less than 4.55 mm·Hg. It was noteworthy to mention that the SD of the errors for MK-EE, MK-BH, dMK-BH, and M-M was within 8 mm·Hg for SBP and DBP. It was consistent with the AAMI requirements of 5 ± 8 mm·Hg (mean ± SD) for BP estimated error [26].

### 4.3. Statistical Analysis

**Figure 7:**
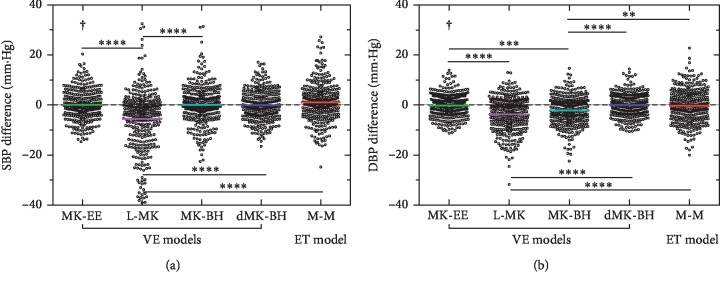
Scatter plot of differences among the five models. Note: significant differences between different PAT models were identified as follows: ^*∗*^*p* < 0.05, ^*∗∗*^*p* < 0.01, ^*∗∗∗*^*p* < 0.001, and ^*∗∗∗∗*^*p* < 0.0001. “**†**”indicates the recommended BP estimation model.

Differences were tested with the Kruskal–Wallis tests and with Dunn's multiple comparison tests to determine whether statistically significant differences were observed between the mean errors of the ET model and the VE models, i.e., L-MK, MK-BH, MK-EE, and dMK-BH, as shown in Figure 7.

In Figure 7, there was a significant difference between the linear model and the nonlinear model in terms of estimating SBP. Similarly, a significant difference between the MK-BH model and the other nonlinear estimation models was also found during estimating DBP. It was noteworthy that MK-BH and M-M showed weaker significant changes. Additionally, there were no significant changes in MK-EE and dMK-BH models.

### 4.4. Sensitivity Analysis

**Figure 8:**
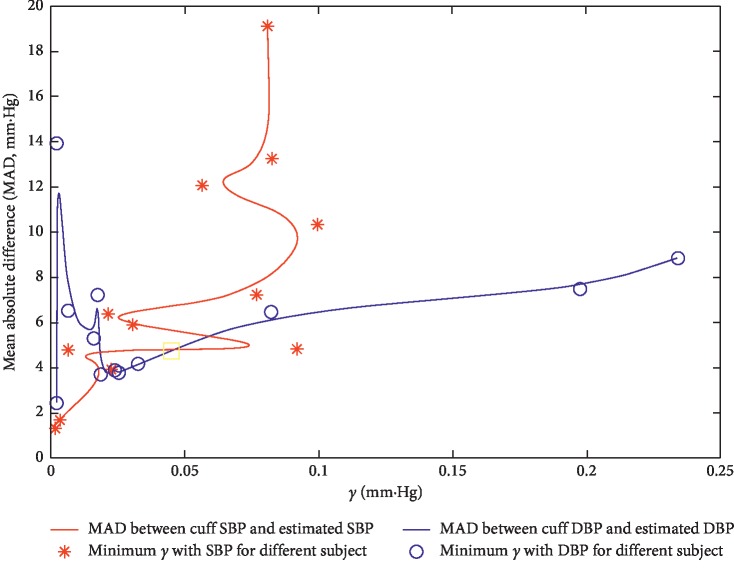
Sensitivity analysis with regard to *γ* for the dMK-BH model. Note: the curves with MAD and *γ* were based on De Boor algorithm [35] to the real-time interpolation for SBP and DBP in all subjects.

As mentioned above, the dMK-BH model with the smallest estimated BP error merited a more in-depth analysis. From its mathematical representation, the determination of *γ*, a vascular information parameter which might be altered with age and the development of cardiovascular diseases, was critical to better estimate BP in long-term ABPM and HBPM. Next, the relationship between MAD and the parameter *γ* (across all 12 subjects over 30,000 heartbeats in Subsection 3.2) was plotted for the dMK-BH model in Figure 8.

In Figure 8, some comparisons could be made. For instance, the minimums of cardiovascular parameter *γ* with SBP and DBP estimation were different for each subject. Moreover, these minimums mainly concentrated in a small range about 0.001–0.03 mm·Hg^−1^ in most subjects. Specifically, the two curves reached the minimums when *γ* were nearby 0.0021 and 0.0239, respectively. Besides, an upward trend approximately with the increase of *γ* between SBP and DBP estimation could be observed after one minimum value (*γ* = 0.0239) in this model based on the experiment. Hence, *γ* was a sensitive cardiovascular parameter for different subjects. In this paper, *γ* was set as 0.031 mm·Hg^−1^ according to Zheng et al.'s report for the healthy subjects (24–35 years old) [9].

## 5. Discussion

In this study, five representative BP-PAT models were studied and optimized based on the same justified and uniform criterion to work out which accurate and practical mode compared with others was well fitted in continuous cuff-less BP monitoring through an implementation of the designated protocol, i.e., the same advanced PTP method. To better evaluate the performance of the five BP-PAT models, the correlation analysis, performance analysis, and statistical analysis were applied.

As for the L-MK, similar to the linear description in the VE models, the BP estimation error was larger than other models. Indeed, this model neglected the complex regulation of the cardiovascular system. There was no explanation for phenomena such as subject's BP fluctuations alternately throughout the day. Besides, it was also not a bound function and not always positive. These performances (see Subsection 4.2) further proved that L-MK was not a good BP estimation method. Usually, linear regression was usually applied for BP estimation, in which the indicators included PAT or extra parameters, such as HR [36], PPG intensity ratio [11, 29], TDB, a kind of arterial stiffness index [36], and other features [19, 37] that could be obtained from ECG and PPG signals. The accuracy of the L-MK model for BP estimation was expected to improve through the combination of PAT and the above indicators by means of multiple regression analysis; however, the computation burden increased sharply.

The MK-BH model, as a VE model, had the lower correlation and the greater estimated error between estimated SBP and cuff SBP (see Tables 3 and 5 and Figure 5) in nonlinear models. For MK-BH, the stroke volume was considered as a constant; however, this parameter varied with the body's demand to oxygen-filled blood, e.g., during exercise [4]. When estimating DBP, the MK-BH model consisted of two parts: a linear function with SBP and a power function without SBP. This suggested that DBP might decrease with an increase in SBP, which was inconsistent with the situation where the SBP varied with the same trend as DBP during the experiment. This study also confirmed that MK-BH was not the better BP estimation model (see Tables 3–5 and Figures 5 and 6) for long-term ABPM and HBPM.

Referring to Esmaili et al.'s report [29], M-M (i.e., the ET model) was good for BP estimation. In contrast to their expectations, this model had the lowest correlation and the greatest estimated error for DBP and SBP, respectively (see Section 4, especially Tables 3–5 and Figures 5 and 6). One reason was that the denominator of the M-M model contained a square root leading to no real solution, which might cause inconvenience in calibration. Another important reason was that the actual arterial system was obviously not a simple tube but rather contained branches, which elastically and geometrically tapered and terminated with the microcirculation [8]. Hence, the M-M model was left for further study to take better account of the influence of the vascular branches in this model.

The dMK-BH model, as a nonlinear VE model and bound function, was based on the Moens–Korteweg equation and the Bramwell–Hill equation (here, MBP as BP). This model, with the strongest correlation between cuff BP and estimated BP and the lowest BP estimated error (see Section 4), was the best BP estimation model because of its rigorous interpretation of physiological parameters (see Table 1). Compared to MK-BH, dMK-BH in BP estimation had a significantly higher accuracy, for which the MBP applied in dMK-BH was a decisive factor. However, it was unsuitable for long-term monitoring due to its complex mathematical relationship about BP vs. PAT including a compound function of power and logarithmic function with *γ* (see Figure 8). Some investigations reported *γ* would change with aging [9, 21, 38] and the development of cardiovascular diseases [33]. It was not easy to obtain an optimal value in different ages and pathophysiologic conditions. Hence, its practicality was limited to an extent.

The nonlinear MK-EE model was conducive to practice due to its rational explanation of physiological information compared to others (see Section 2). In addition, MK-EE, as well as dMK-BH, showed a stronger correlation and lower estimated BP error with both cuff BP and estimated BP than others (see Subsection 4). Furthermore, it could be easily built into wearable sensor devices [23] due to its simple mathematical relationship about BP vs. PAT including a traditional logarithmic function. Recently, the variate of PTT or PWV was suggested for introduction into the MK-EE model for further comprehensive modeling [8, 22].

As mentioned above, the nonlinear dMK-BH model had the strongest correlation and the smallest BP estimation error. Further, there were no significant changes between MK-EE and dMK-BH. Besides, the performance of the MK-EE model showed the best similarity to the dMK-BH model. The MK-BH and M-M, as nonlinear models, could not estimate BP well based on the experiment data, while the MK-EE model and dMK-BH model could estimate BP well (see Section 4). Although the dMK-BH model had the lowest estimated BP error between estimated BP and cuff BP, it needed a further investigation in the practical application since a sensitive cardiovascular parameter (*γ*) was introduced in it. These findings indicated that MK-EE could be a good substitute for dMK-BH in continuous cuff-less BP monitoring. Based on this study, we were confident that the calibration method could be used for ABPM and HBPM to some extent in the future. It was mentioned that periodic calibration should be considered to improve the reliability of BP measurement since the period between calibrations was short and might possibly affect the accuracy of parameters. Recently, Mukkamala and Hahn proposed predictions on the maximum calibration period and acceptable error limits during different ages and genders [28]. Additionally, some research had proposed several methods to improve calibration accuracy. For instance, the covariates were also introduced into calibration methods to better predict BPs, e.g., HR [39], PWV [13], and PIR [11, 29].

The Moens–Korteweg equation has provided a mathematical foundation for advancing research towards the direction of noninvasive BP monitoring. It should be noted that the practical use of the equation implies several assumptions (see Figure 1), which might be invalid for complex behavior and for regulation of the involved arterial tree, such as the thickness-to-radius ratio [24] seen as a constant. Additionally, arterial segments involved in BP estimation were formed for both elastic and muscular arteries, with different biomechanical properties. The influence of these factors on BP estimation needed further study.

## 6. Conclusion and Future Work

In this study, five most popular BP estimation models were investigated and optimized based on PAT under the same advanced PTP method for the first time. The investigation revealed that the MK-EE and dMK-BH, as two VE models based on the Moens–Korteweg equation, were more efficient than the ET model based on the conservation of mass and momentum equation. Considering that the change of human BP was affected by many physiological factors and manifests as a complex nonlinear system, the L-MK with the largest estimated BP error among VE models was not a good choice for ABPM. For family long-term ABPM or HBPM, we suggested selecting MK-EE, a type of VE model, as both cuff BP and estimated BP in this model had stronger correlation and lower estimated BP error than others.

One of the limitations of this investigation was the fact that subjects engaged in the present experiment were generally young and healthy volunteers rather than patients with cardiovascular disease. Thus, further studies with extensive validation that included a larger population of individuals recruited from different age groups and with various pathophysiologies were needed to confirm these outcomes. In addition, the BP-PAT models did not take into account the influence of the pre-ejection period (PEP) and vascular tone changes due to the difficulties in quantitative measuring PEP and vascular tone in ambulatory settings. Hence, a new model including the description of PEP was worth to be established in the future work.

## Figures and Tables

**Table 1:** Summary of mathematical models to calculate BP from PAT.

Models	SBP	DBP	Category and mechanism (linear or nonlinear)	
MK-EE [22, 23]	*a*_1_ *∗* ln PAT+*b*_1_	*a*_1_′*∗*ln PAT+*b*_1_′	Nonlinear	Vascular elasticity (VE) models
L-MK [21]	*a*_2_+*b*_2_*∗*PAT	*a*_2_′+*b*_2_′*∗*PAT	Linear	
MK-BH [4]	SBP_0_ − (2/(*γ∗*PAT_0_))*∗*(PAT − PAT_0_)	SBP − PP_0_*∗*(PAT_0_/PAT)^2^	Nonlinear	
dMK-BH [9]	DBP+PP_0_*∗*(PAT_0_/PAT)^2^	MBP_0_+(2/*γ*)ln(PAT_0_/PAT) − (PP_0_/3)*∗*(PAT_0_/PAT)^2^	Nonlinear	
M-M [29]	$a_{3}+\sqrt{b_{3}+c_{3}*\left(1/{\text{PAT}}^{2}\right)}$	$a_{3}^{\prime }+\sqrt{b_{3}^{\prime }+c_{3}^{\prime }*\left(1/{\text{PAT}}^{2}\right)}$	Nonlinear	Elastic tube (ET) model

*Note*. *γ*  denoted a vascular information parameter which might be altered with age and the development of cardiovascular diseases. For the healthy subjects, it was set as 0.031 mm·Hg^−1^ [9]. PP_0_=SBP_0_ − DBP_0_, MBP_0_=(1/3)SBP_0_+(2/3)DBP_0_. SBP_0_,  DBP_0_, PP_0_  could be determined at the beginning of monitoring by calibration using an additional cuff-type BP monitor device (see Subsection 3.2). *a*_*i*_,  *b*_*i*_,  *a*_*i*_′, *b*_*i*_′(*i*=1,  2,  3);  *c*_*i*_,  *c*_*i*_′ were the corresponding function coefficients. *i* was the subscript, and for their calibration method, see Subsection 3.2.

**Table 2:** Characteristics of the subjects.

Selection factor	Number
Total number (M, F)	12 (9, 3)
Age (years)	25.3 ± 4.1
Height (cm)	168.5 ± 7.4
Body mass (kg)	60.4 ± 9.4
BMI (kg/m^2^)	21.2 ± 2.1
SBP (mm·Hg)	118.37 ± 12.95
DBP (mm·Hg)	69.40 ± 8.79

**Table 3:** The correlations between BP_est_ and BP_cuf._

Models	SBP_est_ vs. SBP_cuf_	DBP_est_ vs. DBP_cuf_
MK-EE	**0.8851**	**0.8571**
L-MK	0.5537	0.6831
MK-BH	0.8131	0.7653
dMK-BH	**0.8873**	**0.8611**
M-M	0.8329	0.7350

**Table 4:** Quantitative comparison of the SSE and RMSE for the subject with the largest BP range.

Indexes	SBP					DBP				
	MK-EE	L-MK	MK-BH	dMK-BH	M-M	MK-EE	L-MK	MK-BH	dMK-BH	M-M
SSE	306.51	**11226**	890.08	283.65	798.00	262.50	**2500.1**	256.71	264.53	428.14
RMSE	3.5737	21.628	6.0899	3.4379	5.7663	3.3072	10.206	3.2705	3.3200	4.2236
^*∗*^CV	22.593	**1.2544**	9.2495	951.70	16.500	27.954	**1.2763**	513.98	35.204	9.4641

*Note*. ^*∗*^CV, a standardized measure of dispersion of a probability distribution or frequency distribution, denoted the coefficient of variation.

**Table 5:** The estimated BP errors in different BP models.

Models	Systolic blood pressure (SBP)		Diastolic blood pressure (DBP)	
	Mean ± SD (mm·Hg)	MAD (mm·Hg)	Mean ± SD (mm·Hg)	MAD (mm·Hg)
**MK-EE**	**0.07** **±** **5.87**	**4.46**	**−0.13** **±** **4.54**	**3.58**
L-MK	**−5.89** **±** **12.74**	**9.34**	**−3.72** **±** **6.79**	**5.91**
MK-BH	0.11 ± 7.53	5.48	−2.10 ± 5.69	4.69
**dMK-BH**	**−0.01** **±** **5.90**	**4.55**	**0.04** **±** **4.40**	**3.38**
M-M	1.11 ± 7.51	5.57	−0.23 ± 6.47	5.13
ANSI/AAMI	|mean| ≤ 5 mm·Hg	≤7 mm·Hg	|mean| ≤ 5 mm·Hg	≤7 mm·Hg
SP10 standard	|SD| ≤ 8 mm·Hg		|SD| ≤ 8 mm·Hg	

## Data Availability

The data used to support the findings of this study are available from the corresponding author upon request.
